# Expression and clinical significance of survivin in ovarian cancer: A meta-analysis

**DOI:** 10.1371/journal.pone.0194463

**Published:** 2018-05-24

**Authors:** Xiaoyan He, Kehu Yang, Hailin Wang, Xiaohong Chen, Huifang Wu, Liang Yao, Shouye Ma

**Affiliations:** 1 Obstetrics and Gynecology Department, Gansu Provincial Hospital, Lanzhou, Gansu, China; 2 The Institute of Clinical Study and Evidence Based Medicine, Gansu Provincial Hospital, Lanzhou, Gansu, China; 3 Evidence-Based Medicine Center and Key Laboratory of Evidence Based Medicine and Knowledge Translation of Gansu Province, College of Basic Medicine, Lanzhou University, Lanzhou, Gansu, China; University of Alabama at Birmingham, UNITED STATES

## Abstract

To assess the clinicopathological significance of survivin in ovarian carcinoma through this meta-analysis. PubMed, EMBASE, Web of Science, and The Cochrane Library databases were searched for relevant studies published through September, 2017. Included studies reported the case-control study of surviving expression with ovarian cancer and its clinicopathological characteristics. The quality assessment was performed according to the Newcastle–Ottawa Scale (NOS) for quality assessment of case–control studies. Statistical analysis was performed with the software Stata 12.0. Twelve eligible studies with a total of 1097 patients were included in this meta-analysis. Survivin overexpression was closely related to FIGO stage (I-II vs. III-IV) of ovarian carcinoma (odds ratio [OR] = 0.26,95% confidence interval [CI]:0.16,0.42),P<0.00001),tumor grade (G1-G2 vs. G3) (OR = 0.29,95%CI(0.17, 0.51),P <0.0001), but was not significantly associated with lymphatic metastasis (OR = 1.53, 95%CI(0.77, 3.03, P = 0.23),ascites (OR = 0.89,95%CI(0.39,2.05),P = 0.79). Our meta-analysis shows that survivin is strongly associated with FIGO stage and tumor grade of ovarian carcinoma. Maybe survivin is a novel clinicopathological marker of ovarian carcinoma.

## Introduction

Presently, ovarian cancer still remains the most fatal cancer of the female reproductive tract [1] due to vague symptomatology and the absence of reliable screening tests in the early stages[2]. The majority of these patients are diagnosed when treatment options are limited, and the overall 5-year survival rates do not exceed 30% [3]. Survivin is a member of the inhibitors of the apoptosis protein (IAP) family, and undetectable in normal adult tissues but highly expressed in several types of cancer, with a potential involvement in malignant transformation and tumor growth [4, 5]. High expression levels of this antiapoptotic protein have been previously found and shown to predict poorer prognosis and shorter survival in a wide range of human cancers, including the gastrocolic carcinoma [6, 7], breast cancer [8], lung [9], and ovary cancer [10, 11]. Thus, survivin is considered a novel clinicopathological marker for numerous human malignant tumors and may be an important prognostic marker in cancer. However, the clinicopathological features associated with survivin expression in ovarian carcinoma remain controversial. To more precisely evaluate the relationship between surviving expression and clinicopathological outcome in ovarian carcinoma, we conducted a meta-analysis of 12 published studies.

## Methods

### Search strategy

We systematically searched PubMed, EMBASE, Web of Science, and The Cochrane Library databases for studies in humans on survivin expression with ovarian cancer and its clinicopathological characteristics, which were published from inception to September, 2017. Computer searches used combinations of subject headings or other key words by the following search strategy: (Ovarian tumor OR Ovarian cancer OR Ovarian carcinoma OR Ovarian malignancy OR Ovarian Neoplasms) AND (Survivin). PubMed database was searched as following: #1 Ovarian tumor[Title/Abstract] OR Ovarian cancer[Title/Abstract] OR Ovarian carcinoma[Title/Abstract] OR Ovarian malignancy[Title/Abstract] OR Ovarian Neoplasms[Title/Abstract] #2 "Ovarian Neoplasms"[Mesh] #3 #1 OR #2 #4 Survivin [Title/Abstract] #5 “Survivin”[Mesh] #6 #4 OR #5 #7 #3 AND #6.

### Study selection

Included studies must meet the following criteria: (1) case-control study focus on the association between the survivin expression and ovarian cancer and its clinicopathological variables;(2)immunohistochemistry(IHC) or real-time PCR (RT-PCR) analysis to evaluate survivin expression in ovarian carcinoma; (3) None of the patients received radiotherapy, chemotherapy or tumor drug treatment before surgery; (4) All cases were not limited with race, nationality or age. The following studies were excluded: (1) conference abstracts, letters, reviews, case reports, commentaries, or expert opinions; (2) studies with insufficient information on clinicopathological characteristics.

### Quality assessment

Study quality was independently assessed by two investigators according to the Newcastle-Ottawa Scale (NOS) for quality assessment of case–control studies [12]. The criteria were quality of selection, comparability, exposure and outcome; the scales allocate stars, with a maximum of nine. The study was evaluated as low quality when the score ≤5 and excluded from our study, by contrast, the study was evaluated as high quality when the score was ≥ 6 and included in our meta-analysis.

### Data extraction

Two investigators independently extracted data that met our inclusion and exclusion criteria. Any discrepancies were resolved by consensus. We extracted the following information: name of the first author, year of publication, country, specific outcomes, total number of individuals, number of cases and controls, clinicopathological characteristics.

### Statistical analysis

Pooled estimates of ORs with 95% CIs were used to evaluate the associations between survivin expression and clinicopathological characteristics of ovarian cancer. Heterogeneity among studies was evaluated with the Cochran Q test and I^2^ statistic [13], inter-study heterogeneity was assumed in cases in which I^2^ >50%, and ORs were pooled according to random-effects models. Alternatively, fixed-effects models were used. Sensitivity analyses evaluated whether the results could have been affected markedly by a single study [14]. In addition, Begg’s funnel plots and Egger’s test were used to statistically assess publication bias, there existed publication bias when p-value less than 0.05 [15]. We used STATA version 12.0 for all statistical analyses and to produce the forest plot. A *p*-value less than .05 was considered statistically significant.

## Results

**Fig 1:**
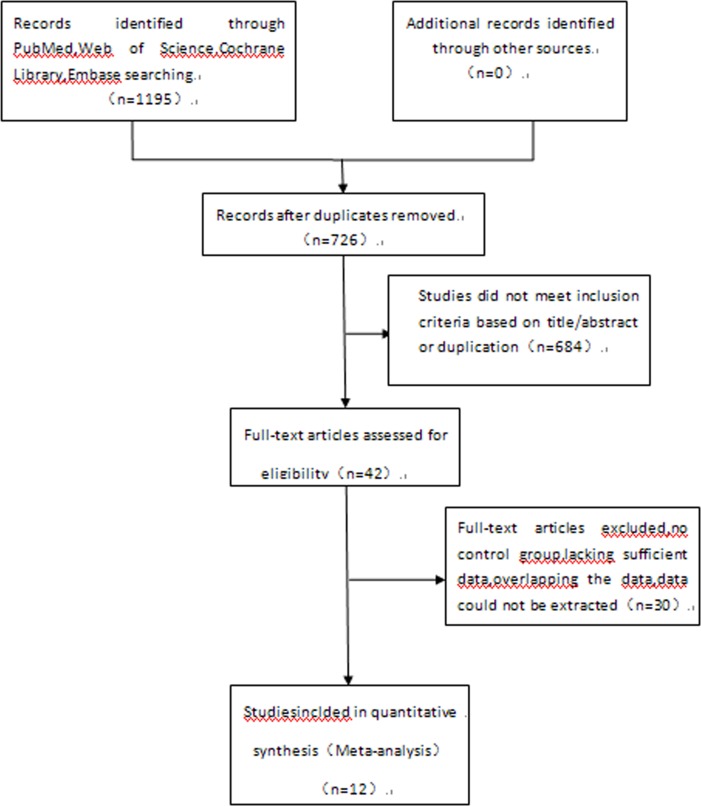
Flow diagram of the study selection process.

The initial search in the electronic databases identified 1195 articles. After excluding duplicate citations and studies that did not meet the inclusion criteria, twelve publications [11, 16–26] were finally included in the meta-analysis (Fig 1). A description of the baseline characteristics are summarized in Table 1. The analysis included 566 ovarian carcinoma cases. Study quality scores (range, 0–9) averaged 7.0, with a proportion of high-quality studies (quality score C8) of 33.3%.

**Table 1:** Characteristics and results of the included studies.

Study	Country	No.of P.(1097)	Method	FIGOStage (Ⅰ-Ⅱ/Ⅲ-Ⅳ)	tumor grade(G1/G2/G3)	lymph nodly metastasis(yes /no)	Survivin(+)				NOS
							Ovarian carcinoma	Borderline ovarian tumor	Ovarian benign tumor	Normal ovarian tissues	
Sui^[^^11^^]^2002	Japan	103	**IHC**	19/28	21/13/13	24/19	24	11	7		**7**
Ju LL^[^^16^^]^2016	China	60	**IHC**	19/20	8/31*		8	3	1		**8**
Kanter M^[^^17^^]^2016	Turkey	98	**IHC**				37	4	3		**8**
Plewka D^[^^18^^]^2015	Europe	157	**IHC**				41	22	13	0	**6**
Turan G^[^^19^^]^2014	Turkey	62	**IHC**				21	10	6		**8**
Qian X^[^^20^^]^2011	China	91	**IHC**				55	4	0		**7**
Huang Y^[^^21^^]^2011	China	65	**IHC**	7/18	2/23*		26	6	5		**8**
Liguang Z^[^^22^^]^2007	China	114	**RT-PCR**	28/35	30**Δ**/33	34/29	46	9	4	0	**6**
Gao Q^[^^23^^]^2007	China	70	**IHC**	10/36	22**Δ**/24		28		5	0	**7**
Yin RT^[^^24^^]^2006	China	69	**IHC**	10/28	13**Δ**/25	18/13	29	9		0	**7**
Ma XY^[^^25^^]^2006	China	143	**IHC**	41/43	32/34/18		53		12	0	**7**
Zhang SL^[^^26^^]^2003	China	65	**RT-PCR**	14/21	24**Δ**/11	13/22	29	8	2	0	**6**

*, G2-G3

**Δ**, G1-G2

No. of P, number of patients; NOS, Newcastle-Ottawa quality assessment scale; RT-PCR, reverse transcription polymerase chain reaction; IHC, immunohistochemistry

### Ovarian carcinoma vs normal ovarian tissues

**Fig 2:**
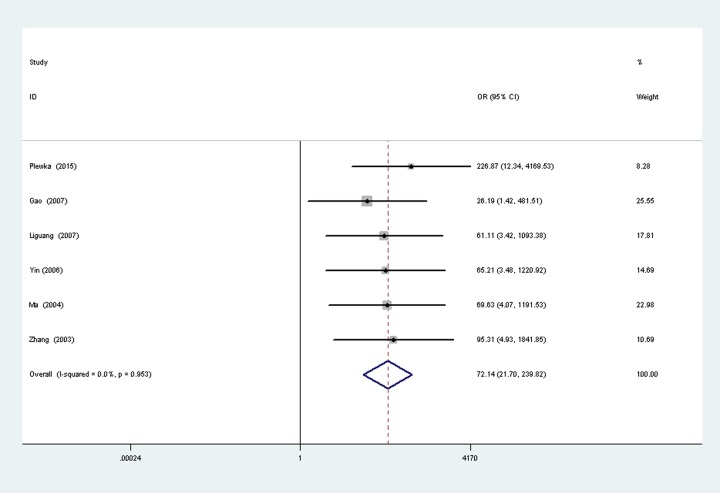
Forest plot depiction of survivin expression and odds ratio (OR) for ovarian carcinoma vs normal ovarian tissues.

A total of six studies [18, 22–26] reported the survivin expression in ovarian cancer vs normal ovarian tissues, with 314 cases of ovarian cancer patients and 79 normal women. There was no significant heterogeneity between the two groups (P = 0.95, I^2^ = 0.0%), so fixed-effects models was used to analysis (Fig 2). Results show that the survivin expression in ovarian cancer is higher than normal group, the difference was statistically significant (OR = 72.14, 95% CI (21.70, 21.70), P < 0.00001).

### Ovarian carcinoma vs ovarian benign tumor

**Fig 3:**
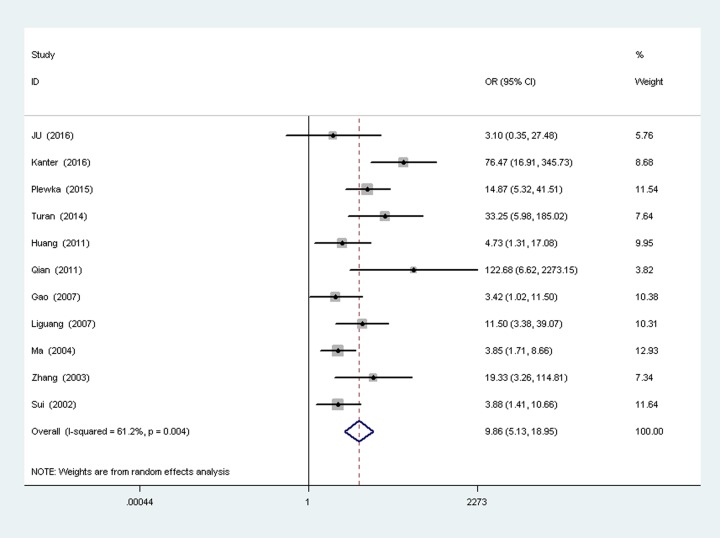
Forest plot depiction of survivin expression and odds ratio (OR) for ovarian carcinoma vs ovarian benign tumor.

A total of eleven studies [11, 16–23, 25, 26] reported the survivin expression in ovarian cancer vs ovarian benign tumor, with 528 cases of ovarian cancer patients and 262 ovarian benign tumor patients. There was heterogeneity between the two groups (*P* = 0.004,I 2 = 61.2%), so random-effects models was used to analysis (Fig 3). Results show that the surviving expression in ovarian cancer is higher than ovarian benign tumor, the difference was statistically significant (OR = 9.86, 95%CI(5.13,18.95), P<0.00001).

### Ovarian carcinoma vs borderline ovarian tumor

**Fig 4:**
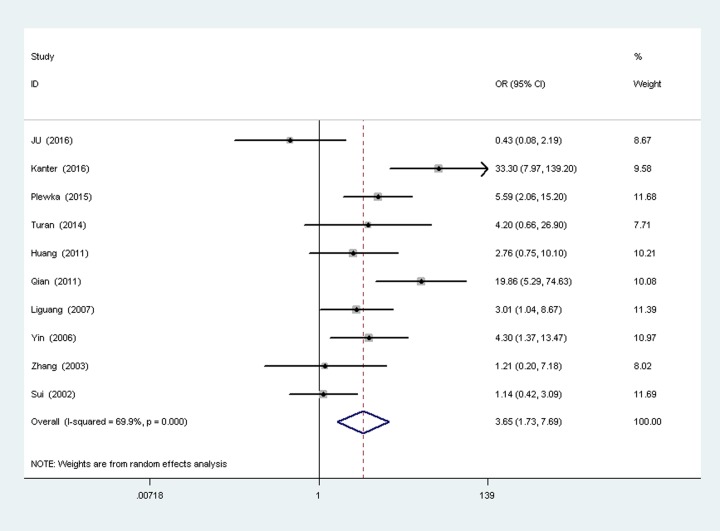
Forest plot depiction of survivin expression and odds ratio (OR) for ovarian carcinoma vs borderline ovarian tumor.

A total of ten studies [11, 16–22, 24, 26] reported the survivin expression in ovarian cancer vs borderline ovarian tumor, with 436 cases of ovarian cancer patients and 190 ovarian borderline tumor patients. There was heterogeneity between the two groups (P = 0.0000,I^2^ = 69.9%), so random-effects models was used to analysis (Fig 4). Results show that the surviving expression in ovarian cancer is higher than borderline ovarian tumor, the difference was statistically significant (OR = 3.65,9%CI(1.73,7.69), P = 0.000).

### Survivin expression with clinicopathological characteristics

**Fig 5:**
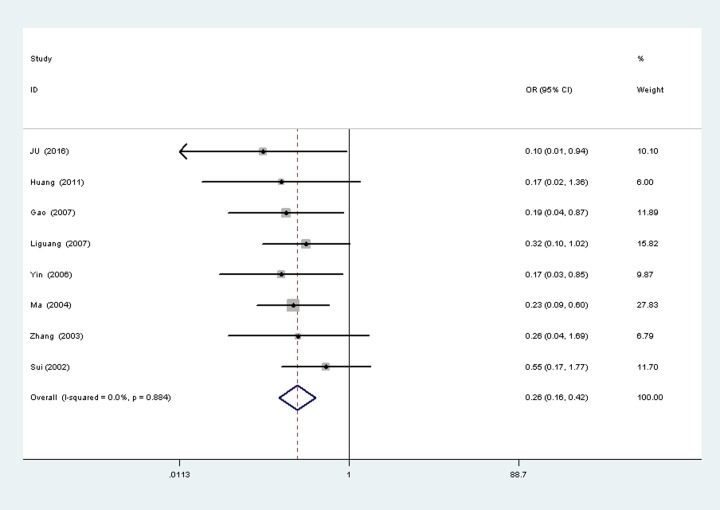
Tumor FIGO stage.

Survivin expression with FIGO stage A total of eight studies [11, 16, 21–26] reported the surviving expression in different FIGO stage of ovarian cancer, with 148 cases of Ⅰ-Ⅱ and cases of Ⅲ- Ⅳ. There was no significant heterogeneity between the two groups (P = 0.884,I^2^ = 0%), so fixed-effects models was used to analysis (Fig 5). Results show that the surviving Expression in Ⅰ-Ⅱ is higher than Ⅲ- Ⅳ, the difference was statistically significant (OR = 0.26, 95%CI(0.16,0.42), P<0.00001).

### Survivin expression with tumor grade

**Fig 6:**
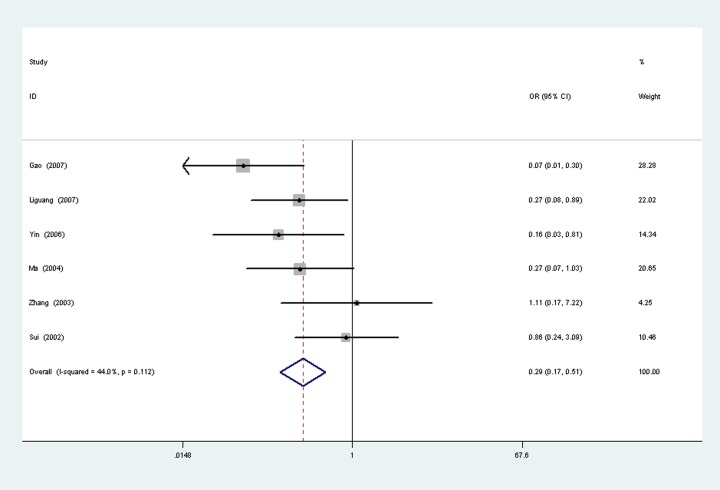
Tumor grade.

A total of six studies [11, 22–26] reported the survivin expression in different tumor grade of ovarian cancer, with 189 cases of G1-G2 and 124 cases of G3. There was no significant heterogeneity between the two groups (P = 0.112,I 2 = 44%), so fixed-effects models was used to analysis (Fig 6). Results show that the survivin expression in G1-G2 is lower than G3, the difference was statistically significant (OR = 0.29,95%CI(0.17,0.51), P<0.0001).

### Survivin expression with lymphatic metastasis

**Fig 7:**
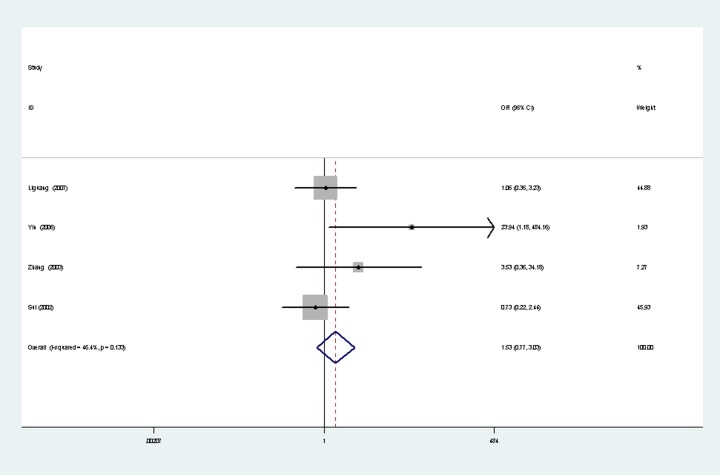
Lymphatic metastasis.

A total of four studies [11, 22, 24, 26] reported the survivin expression in lymphatic metastasis of ovarian cancer, with 89 cases of metastasis and 83 cases of non-metastasis. There was no significant heterogeneity between the two groups (*P* = 0.133, I^2^ = 46.4%), so fixed-effects models was used to analysis (Fig 7). Results show that survivin expression was not significantly associated with lymphatic metastasis (OR = 1.53,95%CI(0.77, 3.03, *P* = 0.23).

### Sensitivity analysis

A sensitivity analysis omitting one study at a time and calculating the pooled ORs for the remainder of the studies in ovarian cancer vs ovarian benign tumor showed that the studies by Kanter et al.[17], Qian et al. [20] and Ma et al.[25] substantially influenced the pooled risk estimates. By excluding these three studies, heterogeneity decreased dramatically (I^2^ = 33.4%; Table 2). In ovarian carcinoma vs borderline ovarian tumor, the studies by JU et al.[16], Kanter etal. [17], Qian et al.[20] and Sui et al. [11] substantially influenced the pooled risk estimates. By excluding these four studies, heterogeneity decreased dramatically (I^2^ = 0%; Table 2).

**Table 2:** Sensitivity analyses.

Groups	Studies(n)	OR (95%CI)	heterogeneity test	
			P	I^2^(%)
Ovarian cancer vs Ovarian benign tumor				
All studies	11	9.86(5.13–18.95)	0.004	61.2
Omitting Kanter M[17]	10	7.25(4.95–10.61)	0.057	45.5
Omitting Qian X^[^^20^^]^	10	8.85(4.68–16.72)	0.008	59.5
Omitting Ma XY^[^^25^^]^	10	11.36(5.60–23.07)	0.010	58.5
Omitting Kanter M[17],Qian X^[^^20^^]^,Ma XY^[^^25^^]^	8	7.66(4.88–12.02)	0.161	33.4
Ovarian cancer vs Borderline ovarian tumor				
All studies	10	3.65(1.73–7.69)	0.000	69.9
Omitting Ju LL^[^^16^^]^	9	4.46(2.18–9.10)	0.004	65.1
Omitting Kanter M[17]	9	2.92(1.49–5.73)	0.011	59.8
Omitting Qian X^[^^20^^]^	9	3.03(1.46–6.28)	0.003	65.2
Omitting Sui^[^^11^^]^	9	4.27(1.98–9.21)	0.003	66.3
Omitting Ju LL^[^^16^^]^, Kanter M[17],QianX^[^^20^^]^, and Sui^[^^11^^]^	6	3.58(2.18–5.90)	0.765	0.0

### Publication bias

**Fig 8:**
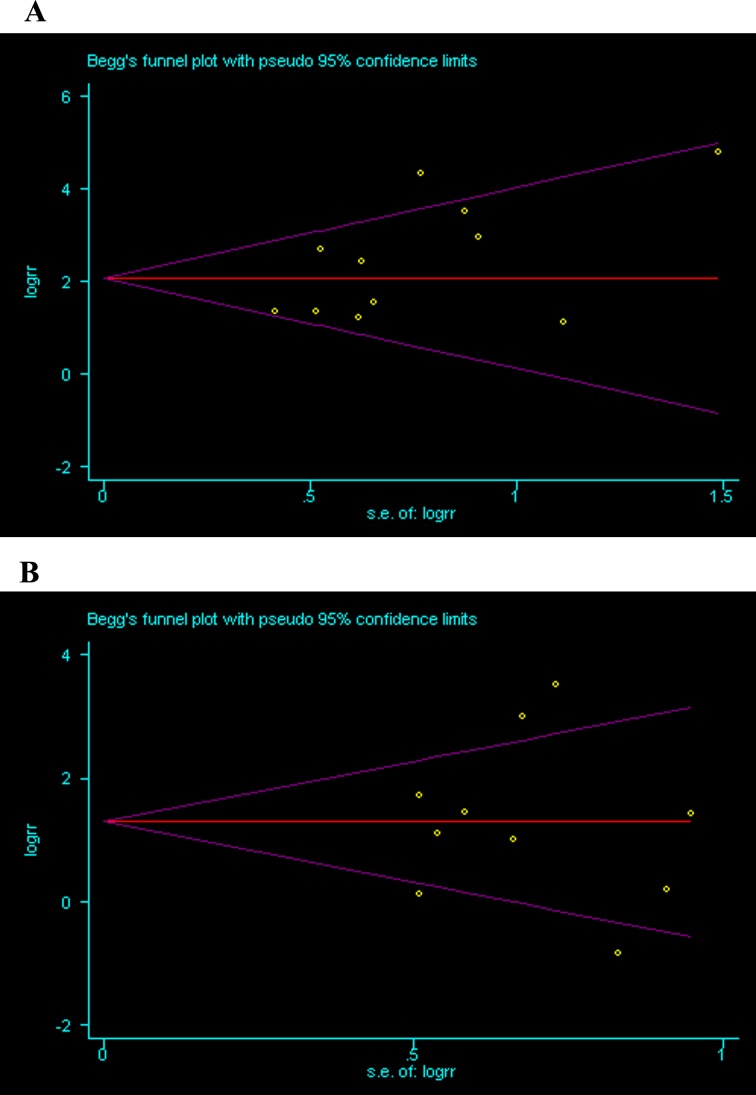
**A** Ovarian carcinoma vs ovarian benign tumor, **B** ovarian carcinoma vs borderline ovarian tumor.

Begg’s funnelplots and Egger’s test were used to assess publication bias in the meta-analysis. There was no indication for publication bias in the Egger’s test of ovarian carcinoma vs ovarian benign tumor (P = 0.073) and ovarian carcinoma vs borderline ovarian tumor (P = 1.000) (Fig 8A and 8B).

## Discussion

Ovarian cancer is one of the most common female cancers and the most frequent cause of gynecologic cancer-related deaths in the word. Hence, presence of biomarkers for the diagnosis, treatment and prediction of prognosis of ovarian neoplasms might guide the management of these patients. Survivin overexpression was found to be related with the aggressiveness of the tumor[27]. The use of survivin expression as a clinicopathological marker of malignancy has received increasing attention. Over expression of survivin has been observed in esophageal, gastric, lung and cervical cancer tissues and represents a poor prognostic factor in these cancer patients [28–32]. Inhibition of apoptosis by survivin is associated with the mitotic spindle assembly checkpoint [33, 34]. Thus, survivin may be a potential target in the treatment of epithelial ovarian cancers. However, despite presence of many studies, definitive role of survivin in the progress of ovarian cancer remains unknown [10, 11, 20, 35] In this study, we investigated the expression and clinical significance of survivin in ovarian cancer in order to find the relationship between them. We found that survivin expression was positively correlated with the FIGO stage and tumor grade. These findings may implicate that survivin has a possible role in malignant potential of ovarian neoplasms. The results of quality evaluation of included studies in our meta-analysis showed that the average of NOS score was 7, and no study was evaluated less than 5, which illustrated that the quality of included studies in our meta-analysis were high. Our meta-analysis results indicate that, survivin expression in the ovarian cancer group is significantly higher than in the normal tissues and benign ovarian tumors (the OR value are as high as 72.14 and 9.86 respectively), with significant difference (P < 0.00001). In normal ovarian tissues and benign ovarian tumor could hardly detect survivin, indicate that survivin has not been activated, survivin was not involved in apoptosis regulation of normal ovarian cells and benign ovarian tissues. However, survivin expressed in ovarian cancer stablely, abnormal expression of survivin may result in the occurrence of ovarian cancer by inhibiting apoptosis. Survivin expression in ovarian cancer is closely related to FIGO stage, tumor grade. The differences of I—II compared with III—IV, G1-G2 compared with G3 were statistically significant (OR = 0.26, OR = 0.29 respectively, P < 0.00001). This suggest the later clinical stage, lower histological differentiation degree, the higher survivin positive expression. Survivin is correlated with the malignant degree of ovarian cancer. Altieri [36] found that survivin over expression was related to tumor metastasis. Zhou [37] found that survivin could promote the transfer of corpus cancer, but our study shows that survivin positive rate between lymph node metastasis and non-lymph metastasis was no statistically different (OR = 1.53, 95%CI(0.77,3.03, P = 0.23,P>0.05). This may be relevant to the different tumor types, or insufficiency of the number of research, or the various different experiment conditions, or the judgment of positive deviation. However, there were limitations to this meta-analysis. First, the techniques used to detect survivin may have differed between the included studies. We determined that IHC and RT-PCR are equally important for the detection of survivin. Second, the survivin antibodies used in the included studies do not discriminate survivin isoforms, so the results may reflect increases in total survivin levels. We suggest that these preliminary findings warrant further analyses in the future. Third, there is the possibility of publication bias, because small studies with null results tend not to be published, as well as outcome reporting biases. In conclusion, despite the above limitations, our meta-analysis supports that survivin overexpression is associated with FIGO stage and tumor grade of ovarian cancer. However, larger clinical studies must be performed to more thoroughly investigate the precise clinicopathological features associated with survivin.

## Supporting information

S1 File PRISMA 2009 checklist.doc and “PRISMA 2009 flow diagram.doc”. (ZIP)
