# Supplementary material for: Expression and clinical significance of survivin in ovarian cancer: A meta-analysis
Source: PLoS One. 2018 May 24;13(5):e0194463. doi: 10.1371/journal.pone.0194463 (PMC5993116; doi:10.1371/journal.pone.0194463)
Supplement: S1 File — (ZIP) [file pone.0194463.s001.zip › PRISMA 2009 flow diagram.doc]

**PRISMA 2009 Flow Diagram**

**Screening**

**Included**

**Eligibility**

**Identification**

Records identified through database searching
(n =1195)

Additional records identified through other sources
(n =0)

Records after duplicates removed
(n = 726)

Records screened
(n =726)

Records excluded
(n =684)

Full-text articles assessed for eligibility
(n =42)

Full-text articles excluded, with reasons
(n =30)

Studies included in qualitative synthesis
(n =12)

Studies included in quantitative synthesis (meta-analysis)
(n =12)
