# Supplementary material for: Expression and clinical significance of survivin in ovarian cancer: A meta-analysis
Source: PLoS One. 2018 May 24;13(5):e0194463. doi: 10.1371/journal.pone.0194463 (PMC5993116; doi:10.1371/journal.pone.0194463)
Supplement: S1 File — (ZIP) [file pone.0194463.s001.zip › PRISMA 2009 checklist.doc]

| **Section/topic** | **#** | **Checklist item** | **Reported on page #** |
| --- | --- | --- | --- |
| **TITLE** | | |  |
| Title | 1 | Identify the report as a systematic review, meta-analysis, or both. | **Expression and Clinical Significance of Survivin in Ovarian Cancer:A meta-analysis** |
| **ABSTRACT** | | |  |
| Structured summary | 2 | Provide a structured summary including, as applicable: background; objectives; data sources; study eligibility criteria, participants, and interventions; study appraisal and synthesis methods; results; limitations; conclusions and implications of key findings; systematic review registration number. | **Abstract**  **Background**  Survivin is considered a novel clinicopathological marker for numerous human malignant tumors and may be an important prognostic marker in cancer. However, the clinicopathological features associated with survivin expression in ovarian carcinoma remain controversial. To more precisely evaluate the relationship between survivin expression and clinicopathological outcome in ovarian carcinoma,we conducteda meta-analysis of 12 published studies.  **Objective**  To assess the clinicopathological significance of survivin in ovarian carcinoma through this meta-analysis.  **Methods**  PubMed,EMBASE,Web of Science,and The Cochrane Library databases were searched for relevant studies published through September, 2017. Included studies reported the case-control study of survivin expression with ovarian cancer and its clinicopathological characteristics. The quality assessment was performed according to the Newcastle–Ottawa Scale（NOS） for quality assessment of case–control studies. Statistical analysis was performed with the software Stata 12.0.  **Results**  Twelve eligible studies with a total of 1097 patients were included in this meta-analysis. Survivin overexpression was closely related to FIGO stage (I-II vs. III-IV) of ovarian carcinoma (odds ratio [OR] =0.26, 95% confidence interval [CI]: 0.16,0.42）,P<0.00001),tumor grade (G1-G2vs. G3) (OR=0.29,95%CI（0.17, 0.51）,P<0.0001)，but was not significantly associated with lymphatic metastasis (OR=1.53,95%CI（0.77,3.03）,P=0.23),ascites (OR=0.89,95%CI（0.39,2.05）,P=0.79).  **Conclusions**  Our meta-analysis shows that survivin is strongly associated with FIGO stage and tumor grade of ovarian carcinoma. [Moreover](javascript:void(0);), survivin is a novel clinicopathological marker of ovarian carcinoma and thus may be a therapeutic target for ovarian carcinoma. |
| **INTRODUCTION** | | |  |
| Rationale | 3 | Describe the rationale for the review in the context of what is already known. | Presently,ovarian cancer still remains the most fatal cancer of the female reproductive tract due to vague symptomatology and the absence of reliable screening tests in the early stages. The majority of these patients are diagnosed when treatment options are limited, and the overall 5-year survival rates do not exceed 30% .  Survivin is a member of the inhibitors of the apoptosis protein (IAP) family, and undetectable in normal adult tissues but highly expressed in several types of cancer,with a potential involvement in malignant transformation and tumor growth.High expression levels of this antiapoptotic protein have been previously found and shown to predict poorer prognosis and shorter survival in a wide range of human cancers, including the gastrocolic carcinoma, breast cancer, lung,and ovary cancer. Thus, survivin is considered a novel clinicopathological marker for numerous human malignant tumors and may be an important prognostic marker in cancer.  However, the clinicopathological features associated with survivin expression in ovarian carcinoma remain controversial. To more precisely evaluate the relationship between survivin expression and clinicopathological outcome in ovarian carcinoma,we conducteda meta-analysis of 12 published studies. |
| Objectives | 4 | Provide an explicit statement of questions being addressed with reference to participants, interventions, comparisons, outcomes, and study design (PICOS). | **P:**Ovarian Cancer;  **I:**-;  **C:** Ovarian carcinoma vs Borderline ovarian tumor vs Ovarian benign tumor  vs [Normal](javascript:void(0);) ovarian tissues;  **O:** Expression of Survivin;  **S:** case-control study |
| **METHODS** | | |  |
| Protocol and registration | 5 | Indicate if a review protocol exists, if and where it can be accessed (e.g., Web address), and, if available, provide registration information including registration number. | There exists no review protocol. |
| Eligibility criteria | 6 | Specify study characteristics (e.g., PICOS, length of follow-up) and report characteristics (e.g., years considered, language, publication status) used as criteria for eligibility, giving rationale. | Included studies must meet the following criteria: (1) case-control study focus on the association between the survivin expression and ovarian cancer and its clinicopathological variables;(2)immunohistochemistry(IHC) or real-time PCR (RT-PCR) analysis to evaluate surviving expression in ovarian carcinoma; (3) None of the patients received radiotherapy ,chemotherapy or tumor drug treatment before surgery; (4) All cases were not limited with race, nationality or age .  The following studies were excluded:(1) conference abstracts, letters, reviews,case reports, commentaries,or expert opinions; (2) studies with insufficient information on clinicopathological characteristics. |
| Information sources | 7 | Describe all information sources (e.g., databases with dates of coverage, contact with study authors to identify additional studies) in the search and date last searched. | We systematically searched PubMed,EMBASE,Web of Science,and The Cochrane Library databases for studies in humans on survivin expression with ovarian cancer and its clinicopathological characteristics until September, 2017. Computer searches used combinations of subject headings or other key words by the following search strategy: (Ovarian tumor OR Ovarian cancer OR Ovarian carcinoma OR Ovarian malignancy OR Ovarian Neoplasms) AND (Survivin). |
| Search | 8 | Present full electronic search strategy for at least one database, including any limits used, such that it could be repeated. | For example: Search strategy of PubMed  #1 Ovarian tumor 1  #2 Ovarian cancer 2  #3 Ovarian carcinoma 3  #4 Ovarian malignancy 4  #5 Ovarian Neoplasms 5  #6 #1 OR #2 OR #3 OR #4 OR #5  #7 Survivin 6  #8 #6 AND #7 |
| Study selection | 9 | State the process for selecting studies (i.e., screening, eligibility, included in systematic review, and, if applicable, included in the meta-analysis). | Included studies must meet the following criteria: (1) case-control study focus on the association between the survivin expression and ovarian cancer and its clinicopathological variables;(2)immunohistochemistry(IHC) or real-time PCR (RT-PCR) analysis to evaluate surviving expression in ovarian carcinoma; (3) None of the patients received radiotherapy ,chemotherapy or tumor drug treatment before surgery; (4) All cases were not limited with race, nationality or age .  The following studies were excluded:(1) conference abstracts, letters, reviews,case reports, commentaries,or expert opinions; (2) studies with insufficient information on clinicopathological characteristics. |
| Data collection process | 10 | Describe method of data extraction from reports (e.g., piloted forms, independently, in duplicate) and any processes for obtaining and confirming data from investigators. | Two investigators independently extracted data that met our inclusion and exclusion criteria. Any discrepancies were resolved by consensus. |
| Data items | 11 | List and define all variables for which data were sought (e.g., PICOS, funding sources) and any assumptions and simplifications made. | We extracted the following information: name of the first author, year of publication, country, specific outcomes, total number of individuals, number of cases and controls,  clinicopathological characteristics. |
| Risk of bias in individual studies | 12 | Describe methods used for assessing risk of bias of individual studies (including specification of whether this was done at the study or outcome level), and how this information is to be used in any data synthesis. | Sensitivity analyses evaluated whether the results could have been affected markedly by a single study. |
| Summary measures | 13 | State the principal summary measures (e.g., risk ratio, difference in means). | Pooled estimates of ORs with 95% CIs were used to evaluate the associations between survivin expression and clinicopathological characteristics of overian cancer. |
| Synthesis of results | 14 | Describe the methods of handling data and combining results of studies, if done, including measures of consistency (e.g., I^2^) for each meta-analysis. | Heterogeneity among studies was evaluated with the Cochran Q test and I^2^ statistic, inter-study heterogeneity was assumed in cases in which I^2^ >50%, and ORs were pooled according to random-effects models.Alternatively, fixed-effects models were used. |

Page 1 of 2

| **Section/topic** | **#** | **Checklist item** | **Reported on page #** |
| --- | --- | --- | --- |
| Risk of bias across studies | 15 | Specify any assessment of risk of bias that may affect the cumulative evidence (e.g., publication bias, selective reporting within studies). | Begg’s funnel plots and Egger’s test were used to statistically assess publication bias, there existed publication bias when p-value less than 0.05. |
| Additional analyses | 16 | Describe methods of additional analyses (e.g., sensitivity or subgroup analyses, meta-regression), if done, indicating which were pre-specified. | There is no additional analyses. |
| **RESULTS** | | |  |
| Study selection | 17 | Give numbers of studies screened, assessed for eligibility, and included in the review, with reasons for exclusions at each stage, ideally with a flow diagram. | Records identified through PubMed,Web of Science,Cochrane Library,Embase searching  （n=1195）  Additional records identified through other sources  （n=0）  Records after duplicates removed  （n=726）  Studies did not meet inclusion criteria based on title/abstract or duplication（n=684）  Full-text articles assessed for eligibility（n=42）  Full-text articles excluded,no control group,lacking sufficient data,overlapping the data,data could not be extracted（n=30）  Studiesinclded inquantitative  synthesis（Meta-analysis）  （n=12） |
| Study characteristics | 18 | For each study, present characteristics for which data were extracted (e.g., study size, PICOS, follow-up period) and provide the citations. | **Table 1. Characteristics and results of the included studies.** Study \| Country \| No.of P.(1097) \| Method \| FIGOStage (Ⅰ-Ⅱ/Ⅲ-Ⅳ) \| tumor grade(G1/G2/G3) \| lymph nodly metastasis(yes /no) \| Survivin（+） \| \| \| \| NOS; \| \| \| \| \| \| \| Ovarian carcinoma \| Borderline ovarian tumor \| Ovarian benign tumor \| Normal ovarian tissues \|; Sui^[11]^2002 \| Japan \| 103 \| **IHC** \| 19/28 \| 21/13/13 \| 24/19 \| 24 \| 11 \| 7 \| \| **7**; Ju LL^[16]^2016 \| China \| 60 \| **IHC** \| 19/20 \| 8/31* \| \| 8 \| 3 \| 1 \| \| **8**; Kanter M^[17]^2016 \| Turkey \| 98 \| **IHC** \| \| \| \| 37 \| 4 \| 3 \| \| **8**; Plewka D^[18]^2015 \| Europe \| 157 \| **IHC** \| \| \| \| 41 \| 22 \| 13 \| 0 \| **6**; Turan G^[19]^2014 \| Turkey \| 62 \| **IHC** \| \| \| \| 21 \| 10 \| 6 \| \| **8**; Qian X^[20]^2011 \| China \| 91 \| **IHC** \| \| \| \| 55 \| 4 \| 0 \| \| **7**; Huang Y^[21]^2011 \| China \| 65 \| **IHC** \| 7/18 \| 2/23* \| \| 26 \| 6 \| 5 \| \| **8**; Liguang Z^[22]^2007 \| China \| 114 \| **RT-PCR** \| 28/35 \| 30**△**/33 \| 34/29 \| 46 \| 9 \| 4 \| 0 \| **6**; Gao Q^[23]^2007 \| China \| 70 \| **IHC** \| 10/36 \| 22**△**/24 \| \| 28 \| \| 5 \| 0 \| **7**; Yin RT^[24]^2006 \| China \| 69 \| **IHC** \| 10/28 \| 13**△**/25 \| 18/13 \| 29 \| 9 \| \| 0 \| **7**; Ma XY^[25]^2006 \| China \| 143 \| **IHC** \| 41/43 \| 32/34/18 \| \| 53 \| \| 12 \| 0 \| **7**; Zhang SL^[26]^2003 \| China \| 65 \| **RT-PCR** \| 14/21 \| 24**△**/11 \| 13/22 \| 29 \| 8 \| 2 \| 0 \| **6** *，G2-G3；△，G1-G2；No. of P, number of patients; NOS, Newcastle-Ottawa quality assessment scale; RT-PCR, reverse transcription polymerase chain reaction; IHC,immunohistochemistry; |
| Risk of bias within studies | 19 | Present data on risk of bias of each study and, if available, any outcome level assessment (see item 12). | A sensitivity analysis omitting one study at a time and calculating the pooled ORs for the remainder of the studies in ovarian cancer vs ovarian benign tumor showed that the studies by Kanter et al. [16], Qian et al. [19] and Ma et al. [24] substantially influenced the pooled risk estimates. By excluding these three studies, heterogeneity decreased dramatically (I 2 = 33.4%; Table 2). Table 2 Sensitivity analyses Groups \| Studies(n) \| OR (95％CI) \| heterogeneity test \|; \| \| \| Ｐ \| Ｉ^２^(％); Ovarian cancer vs Ovarian benign tumor \| \| \| \|; All studies \| 11 \| 9.86(5.13–18.95) \| 0.004 \| 61.2; Omitting Kanter M[17] \| 10 \| 7.25(4.95–10.61) \| 0.057 \| 45.5; Omitting Qian X^[20]^ \| 10 \| 8.85(4.68–16.72) \| 0.008 \| 59.5; Omitting Ma XY^[25]^ \| 10 \| 11.36(5.60–23.07) \| 0.010 \| 58.5; Omitting Kanter M[17],Qian X^[20]^,Ma XY^[25]^ \| 8 \| 7.66(4.88–12.02) \| 0.161 \| 33.4; Ovarian cancer vs Borderline ovarian tumor \| \| \| \|; All studies \| 10 \| 3.65(1.73–7.69) \| 0.000 \| 69.9; Omitting Ju LL^[16]^ \| 9 \| 4.46(2.18–9.10) \| 0.004 \| 65.1; Omitting Kanter M[17] \| 9 \| 2.92(1.49–5.73) \| 0.011 \| 59.8; Omitting Qian X^[20]^ \| 9 \| 3.03(1.46–6.28) \| 0.003 \| 65.2; Omitting Sui^[11]^ \| 9 \| 4.27(1.98–9.21) \| 0.003 \| 66.3; Omitting Ju LL^[16]^, Kanter M[17],QianX^[20]^, and Sui^[11]^ \| 6 \| 3.58(2.18–5.90) \| 0.765 \| 0.0 |
| Results of individual studies | 20 | For all outcomes considered (benefits or harms), present, for each study: (a) simple summary data for each intervention group (b) effect estimates and confidence intervals, ideally with a forest plot. |   Figure 2- Forest plot depiction of survivin expression and odds ratio (OR) for Ovarian carcinoma vs [Normal](javascript:void(0);) ovarian tissues    Figure 3- Forest plot depiction of survivin expression and odds ratio (OR) for Ovarian carcinoma vs Ovarian benign tumor    Figure 4- Forest plot depiction of survivin expression and odds ratio (OR) for Ovarian carcinoma vs Borderline ovarian tumor    Figure 5- tumor FIGO stage    Figure 6- tumor grade    Figure 7-Lymphatic metastasis |
| Synthesis of results | 21 | Present results of each meta-analysis done, including confidence intervals and measures of consistency. | **Ovarian carcinoma vs** [**Normal**](javascript:void(0);) **ovarian tissues**  A total of six studies [17,21-25] reported the survivin expression in ovarian cancer vs normal ovarian tissues,with 314 cases of ovarian cancer patients and 79 normal women.There was no significant heterogeneity between the two groups (P = 0.95, I^2^= 0.0%), so fixed-effects models was used to analysis (Figure 2). Results show that the survivin expression in ovarian cancer is higher than normal group, the difference was statistically significant (OR = 72.14, 95% CI (21.70, 21.70), P < 0.00001).  **Ovarian carcinoma vs** **Ovarian benign tumor**  A total of [eleven](javascript:void(0);) studies [15-22,24-26] reported the survivin expression in ovarian cancer vs ovarian benign tumor,with 528 cases of ovarian cancer patients and 262 ovarian benign tumor patients. There was heterogeneity between the two groups (P =0.004, I^2^=61.2%), so random-effects models was used to analysis (Figure 3). Results show that the survivin expression in ovarian cancer is higher than ovarian benign tumor, the difference was statistically significant (OR=9.86, 95%CI（5.13,18.95）,P<0.00001).  **Ovarian** **carcinoma vs** **Borderline ovarian tumor**  A total of [ten](javascript:void(0);) studies[15-21,23,25-26] reported the survivin expression in ovarian cancer vs borderline ovarian tumor,with 436 cases of ovarian cancer patients and 190 ovarian borderline tumor patients. There was heterogeneity between the two groups (P =0.0000, I^2^=69.9%), so random-effects models was used to analysis (Figure 4). Results show that the survivin expression in ovarian cancer is higher than borderline ovarian tumor, the difference was statistically significant (OR=3.65,9%CI（1.73,7.69）,P=0.000).  **Survivin expression with Clinicopathological characteristics**  Survivin expression with FIGO stage A total of eight studies[15,20,21-26] reported the survivin expression in different FIGO stage of ovarian cancer,with 148 cases ofⅠ-Ⅱ and 229 cases of Ⅲ - Ⅳ. There was no no significant heterogeneity between the two groups (P=0.884, I^2^=0%), so fixed-effects models was used to analysis (Figure 5). Results show that the survivin expression inⅠ-Ⅱ is higher than Ⅲ - Ⅳ, the difference was statistically significant (OR=0.26,95%CI（0.16， 0.42）,P<0.00001).  Survivin expression with tumor grade A total of six studies[21-26]reported the survivin expression in different tumor grade of ovarian cancer,with 189 cases of G1-G2 and 124 cases of G3. There was no significant heterogeneity between the two groups (P=0.112, I^2^=44%), so fixed-effects models was used to analysis (Figure 6). Results show that the survivin expression in G1-G2 is lower than G3, the difference was statistically significant (OR=0.29,95%CI（0.17, 0.51）,P<0.0001). |
| Risk of bias across studies | 22 | Present results of any assessment of risk of bias across studies (see Item 15). | Begg’s funnelplots and Egger’s test were used to assess publication bias in the meta-analysis.There was no indication for publication bias in the Egger’s test of ovarian carcinoma vs ovarian benign tumor(P = 0.073) and ovarian carcinoma vs borderline ovarian tumor(P = 1.000)( Figure 8A-B).  .   Figure 8A-Ovarian carcinoma vs Ovarian benign tumor    Figure 8B-Ovarian carcinoma vs Borderline ovarian tumor |
| Additional analysis | 23 | Give results of additional analyses, if done (e.g., sensitivity or subgroup analyses, meta-regression [see Item 16]). |  |
| **DISCUSSION** | | |  |
| Summary of evidence | 24 | Summarize the main findings including the strength of evidence for each main outcome; consider their relevance to key groups (e.g., healthcare providers, users, and policy makers). | Twelve eligible studies with a total of 1097 patients were included in this meta-analysis. Survivin overexpression was closely related to FIGO stage (I-II vs. III-IV) of ovarian carcinoma (odds ratio [OR] =0.26, 95% confidence interval [CI]: 0.16,0.42）,P<0.00001),tumor grade (G1-G2vs. G3) (OR=0.29,95%CI（0.17, 0.51）,P<0.0001)，but was not significantly associated with lymphatic metastasis (OR=1.53,95%CI（0.77,3.03）,P=0.23),ascites (OR=0.89,95%CI（0.39,2.05）,P=0.79).Our meta-analysis shows that survivin is strongly associated with FIGO stage and tumor grade of ovarian carcinoma. [Moreover](javascript:void(0);), survivin is a novel clinicopathological marker of ovarian carcinoma and thus may be a therapeutic target for ovarian carcinoma. |
| Limitations | 25 | Discuss limitations at study and outcome level (e.g., risk of bias), and at review-level (e.g., incomplete retrieval of identified research, reporting bias). | However,there were limitations to this meta-analysis. First, the techniques used to detect surviving may have differed between the included studies. We determined that IHC and RT-PCR are equally important for the detectionof survivin. Second, the survivin antibodies used in the included studies do not discriminate survivin isoforms,so the results may reflect increases in total survivin levels.We suggest that these preliminary findings warrant further analyses in the future.Third,there is the possibility of publication bias, because small studies with null results tend not to be published, as well as outcome reporting biases. |
| Conclusions | 26 | Provide a general interpretation of the results in the context of other evidence, and implications for future research. | In conclusion,despite the above limitations,our meta-analysis supports that surviving overexpression is associated with FIGO stage and tumor grade of ovarian cancer. Therefore, surviving may be a therapeutic target for ovarian carcinoma. However, larger clinical studies must be performed to more thoroughly investigate the precise clinicopathological features associated with survivin. |
| **FUNDING** | | |  |
| Funding | 27 | Describe sources of funding for the systematic review and other support (e.g., supply of data); role of funders for the systematic review. | The first author pays all fees. |

*From:* Moher D, Liberati A, Tetzlaff J, Altman DG, The PRISMA Group (2009). Preferred Reporting Items for Systematic Reviews and Meta-Analyses: The PRISMA Statement. PLoS Med 6(7): e1000097. doi:10.1371/journal.pmed1000097

For more information, visit: **www.prisma-statement.org**.

Page 2 of 2
